# Remediation of Allophonic Perception and Visual Attention Span in Developmental Dyslexia: A Joint Assay

**DOI:** 10.3389/fpsyg.2019.01502

**Published:** 2019-07-16

**Authors:** Rachel Zoubrinetzky, Gregory Collet, Marie-Ange Nguyen-Morel, Sylviane Valdois, Willy Serniclaes

**Affiliations:** ^1^Centre Référent des Troubles du Langage et des Apprentissages, Pôle Couple-Enfant, Centre Hospitalier Universitaire, Grenoble, France; ^2^Laboratoire de Psychologie et NeuroCognition, CNRS, UMR 5105, Université Grenoble-Alpes, Grenoble, France; ^3^Unité de Recherche en Neurosciences Cognitives, Centre de Recherches en Cognition et Neurosciences, Université Libre de Bruxelles, Brussels, Belgium; ^4^Institute of Neuroscience and Cognition, CNRS, UMR 8002, Université Sorbonne Paris Cité, Paris, France

**Keywords:** dyslexia, reading, remediation, allophonic perception, visual attention span

## Abstract

Categorical perception of phonemes and visual attention span are cognitive processes that contribute independently to poor reading skills in developmental dyslexia. We here explored whether training programs specifically targeting one or the other process do improve reading performance in dyslexic children. The dyslexic participants were trained using either the RapDys© program designed to improve phonemic perception or the MAEVA© program targeting visual attention span. Each participant was provided the two programs successively for intensive training. Results show specific effects of RapDys© on phonemic discrimination and pseudo-word reading. MAEVA© specifically improved visual attention span and irregular word reading. Phonemic awareness and regular word reading improved after application of both training programs, suggesting similar positive effects of both methods although effects of concomitant phonic training cannot be ruled out (as there was no control group). The overall findings suggest that both categorical perception and visual attention span remediation contribute to reading.

## Introduction

Many studies during the last 40 years have been conducted to explore the cognitive origin of developmental dyslexia. Advances in this research field is thought to be critical to improve the remediation of reading acquisition disorders, assuming that a training program specifically targeting the cognitive deficit at the origin of the reading problem should improve reading skills more than when targeting the symptoms. Dyslexia can possibly arise from a deficit in one of the three basic factors that condition reading acquisition: the visual processing of letters within strings, the phonological processing of speech sounds and the association between letters and phonological units. Behavioral, neurophysiological, and genetic evidence support the contention that dyslexia might arise not only from a phonological deficit (as widely admitted: Snowling, 2001; Vellutino et al., 2004) but also from other deficits that specifically affect either visual or phono-visual processes (Serniclaes and Sprenger-Charolles, 2015).

In the present paper, we will compare the effects of two different remediation methods for dyslexia, a phonological method and a visual one. The “RapDys©” method stems from the hypothesis that the phonological deficit in dyslexia arises from a specific mode of speech perception that is based on allophonic features and segments, rather than phonemic ones (Serniclaes et al., 2004). Allophonic theory locates the origin of the phonological deficit to an unconscious perceptual factor contrary to the classical phonological theories of dyslexia that ascribe such deficit to a lack of phonological “awareness” (i.e., in the conscious access to phonemic units). The “MAEVA©” method stems from the hypothesis that the visual deficit in dyslexia is due to a visual attention (VA) dysfunction that impairs multi-letter parallel processing (Bosse et al., 2007; Lassus-Sangosse et al., 2008). Unlike other visual theories that relate the visual deficit to a deficiency in either the representation of letters (Cohen and Dehaene, 2004) or in the orientation of spatial attention (Franceschini et al., 2012), the VA span theory attributes the visual deficit in dyslexia to a deficit in the simultaneous processing of distinct visual elements.

### Phonological Awareness, Categorical Perception, VA Span, and Dyslexia

The phonological theory covers a wide field of deficits, but the PA deficit is the most robust and the most documented. Longitudinal studies have shown the predictive value of earlier PA skills on later reading development (Castles and Coltheart, 2004; Lervag et al., 2009; Boets et al., 2010; Dandache et al., 2014). A meta-analysis based on 235 studies including conventional comparison studies (control group of the same age), reading-level matched designs and correlational data (Melby-Lervåg et al., 2012), confirms the fundamental role of PA in developmental dyslexia and in learning to read.

Phonological skills may further depend on the quality of auditory processing (Goswami, 2015). Many studies that have examined speech perception in dyslexic individuals have reported a deficit in the categorical perception of phonemes in children with dyslexia (for a review, see Noordenbos and Serniclaes, 2015). The categorical perception deficit is characterized by a reduced discrimination between sounds that straddle the phonemic boundary along some acoustic continuum. According to the allophonic theory, such deficit results from the use of several boundaries along the same continuum, not only the phonemic boundary but also allophonic boundaries. Both behavioral and neuro-physiological data support this theory (Bogliotti et al., 2008; Dufor et al., 2009; Noordenbos et al., 2012, 2013; Serniclaes and Seck, 2018). Different studies have shown that the deficit in categorical perception of phonemic features, e.g., the voicing distinction between /d/ and /t/, is due to enhanced sensitivity to allophonic features, e.g., to the difference between two variants of /d/. The consequence of allophonic perception for reading acquisition is that it disrupts the link between speech sounds and graphemes, even with a completely transparent orthography, as there are several allophones for the same phoneme.

Visual and attentional theories have also been proposed (Hari and Renvall, 2001; Facoetti, 2004; Bosse et al., 2007; Stein, 2014) but their causal relationship with developmental dyslexia is hotly debated (Goswami, 2015). Some visual deficits – those related to a magnocellular deficit (Borsting et al., 1996; Witton et al., 1998; Cestnick and Coltheart, 1999), to temporal attention deficits (Hari and Renvall, 2001; Lallier and Valdois, 2012) or to spatial attention (Facoetti et al., 2006, 2010) – typically co-occur with the phonemic awareness (PA) deficit that is viewed as the core deficit in developmental dyslexia. In contrast, evidence from both group studies (Bosse et al., 2007; Germano et al., 2014; Zoubrinetzky et al., 2014) and case studies (Valdois et al., 2003, 2011; Dubois et al., 2010; Lallier et al., 2010) studies shows that the VA span and PA deficits typically dissociate in children with dyslexia. Previous studies further showed that VA span correlates with reading skills independently of PA in both dyslexic (Bosse et al., 2007; Germano et al., 2014; Zoubrinetzky et al., 2014) and typical readers (Bosse and Valdois, 2009; van den Boer and de Jong, 2018), thus suggesting that the PA and VA span deficits independently affect reading performance.

In a recent study (Zoubrinetzky et al., 2016), we brought first evidence supporting the independence of the categorical perception deficit that relates to PA and the VA span deficit in the dyslexic population. Children with dyslexia who had a PA deficit but preserved VA span were found to exhibit a categorical perception deficit characterized by lower precision of the phonemic boundary, while children with poor VA span but preserved PA did not show such categorical perception deficit. These results strengthen the hypothesis of a specific link between categorical perception and PA while VA span appears as a separate cognitive mechanism. A direct consequence of these findings for remediation is that categorical perception if properly trained should improve PA and reading related skills without affecting VA span. Reversely, a VA span-oriented training should improve VA span without affecting PA and yield significant improvement in reading related skills.

### Phonological Remediation

A meta-analysis of the effects of PA instruction based on 52 studies (Ehri et al., 2001) showed that the methods that include PA training improve reading skills in at risk for reading disability children more than those that do not include such training, but the effect is much stronger when letter-sound associations are simultaneously trained. Studies on children with dyslexia also showed that a program including PA and phoneme discrimination training improved both PA and reading skills (Simos et al., 2002; Eden et al., 2004) but the programs used for training further included exercises with printed syllables and printed words. Thus, we cannot definitely exclude that the reported effects primarily reflected direct improvement of the reading process.

Other training studies focused more specifically on the perception and discrimination of phonemes. Some used the Play-On^®^ software (Danon-Boileau and Barbier, 2002) to train phoneme discrimination through audiovisual exercises in which a CV syllable was orally displayed (/pa/) followed by the presentation of two printed syllables (‘pa’ or ‘ba’), one of which corresponded to the oral target. The intensive use of the Play-On^®^ software resulted in positive effect in reading (Magnan et al., 2004; Magnan and Ecalle, 2006). In another study (Veuillet et al., 2007), the same software was used to train children with dyslexia who were selected to have an allophonic discrimination. Following audiovisual training, categorical perception and reading skills improved, and a change in lateralization of the medial olivo-cochlear system (MOC) of the ascending auditory pathways involved in the mechanisms of speech perception in noise was reported. Although the theoretical background of these studies focused on the phonemic component of the Play-On^®^ program, we cannot ignore the printed material involved. Results were less conclusive when phoneme discrimination was trained without reference to printed material. A positive effect of pure discrimination training on PA and reading skills has been reported for typical readers (Moore et al., 2005), but the effect was only found for PA in dyslexic children, without transfer to reading (Hurford, 1990; for similar results on poor readers: Thomson et al., 2013). We will here explore the effect of RapDys©, a pure phoneme categorical perception training program (without printed material), on PA and reading skills in children with dyslexia.

RapDys© was found useful to improve PA when proposed to children with specific language impairment (Collet et al., 2012). During 18 sessions of about 30 min, children were trained to discriminate two different phonemes within a /dә/-/tә/ continuum. Their ability to identify and discriminate phonemes improved following training and subsequently their PA abilities. These results suggest that categorical perception training positively affects PA skills. However, we lack evidence for transfer to reading as reading skills were not monitored in this study. If we assume that allophonic perception in some children with dyslexia prevents normal PA development and thus normal acquisition of literacy skills, reshaping phonological categories using this specific tool could be useful and have direct and positive effect on reading performance.

### VA Span Remediation

The VA span is a recent construct whose effect on reading was only investigated during the last decade. The VA span typically dissociates from PA skills and relates to distinct brain regions. The superior parietal lobules bilaterally are activated in typical readers when performing VA span tasks (Peyrin et al., 2011; Lobier et al., 2012a) and these regions are hypo-activated in dyslexic individuals with a VA span deficit (Peyrin et al., 2011; Reilhac et al., 2013; Lobier et al., 2014). These cerebral regions are well known for their involvement in the dorsal attentional network (Corbetta and Shulman, 2002; Valdois et al., 2018) but are not involved in the language network (Price, 2012). These findings suggest that different brain networks relate to the VA span and PA deficit in developmental dyslexia (see Peyrin et al., 2012), in support of the independence of the VA span deficit.

Evidence for a link between VA span and reading is well documented and there is strong empirical evidence against a consequence relationship, according to which children would show poor VA span due to their poor reading skills (Lobier and Valdois, 2015, for a review). However, only a few data supports a causal relationship. A single study was carried out to explore whether providing training in VA span would ameliorate reading (Valdois et al., 2014b). In this study, the COREVA^®^ training program (Valdois et al., 2014a) was proposed to a dyslexic child, MP, who showed impaired VA span but normal PA. The program included visual search and visual discrimination tasks, string comparison and visual matching. The stimuli were either verbal or non-verbal and the number of elements varied from one to five elements to be simultaneously processed. The first steps of training required processing a one element target in predominantly non-verbal exercises. The number of elements to be simultaneously processed then progressively increased with a shift from predominantly non-verbal to predominantly verbal material (including graphemes, short orthographic syllables or short words). Following intensive training with COREVA^®^ during 6 weeks, MP’s VA span significantly increased. Amelioration of her reading skills was further reported immediately after training with sustained effects at mid-term, 11 months after the end of the training session. Functional neuroimaging (fMRI) further showed an hypoactivation of the superior parietal lobules prior to the intervention but re-activation of these cerebral regions following VA span training. This study shows positive effect of COREVA^®^ on VA span and reading performance. However, the inclusion of printed material (letters, graphemes or words) in COREVA^®^ challenges the specificity of the effect and prevents establishing an undisputable causal link between VA span and reading acquisition.

### The Present Study

In the present study, we will compare the effects of a new VA span training program, MAEVA©, with those of the RapDys© program (adapted from Collet et al., 2012) on the reading performance of children with dyslexia.^1^ RapDys© does not include any orthographic material and MAEVA© mainly uses non-orthographic stimuli.

If VA span and phoneme categorical perception deficits causally and independently relate to developmental dyslexia, we would expect a positive effect of the two training programs on reading skills. RapDys© should improve not only categorical perception but further PA and reading performance without any significant effect on VA span. Reversely, MAEVA© should improve VA span and reading performance without affecting PA skills.

The dyslexic participants were randomly assigned to one of two training groups (the RapDys-MAEVA vs. MAEVA- RapDys group). A crossover design was adopted so that each training group served as control for the effects of the other group. We expected RapDys© but not MAEVA© to improve phonemic discrimination while MAEVA© but not RapDys© should improve VA span. RapDys© should have larger effects than MAEVA© on PA. With respect to reading subskills, RapDys© should have larger effects than MAEVA© on pseudo-word reading, a reading subskill which closely relates to phonological abilities. Finally, the relative effects of MAEVA© and RapDys© on the other reading subskills (regular word, irregular-word, and text reading) remain open questions.

## Materials and Methods

### Participants

Forty-five children with dyslexia [mean age = 10 years 7 months, standard deviation (SD) = 16 months] participated in this study. All were French native speakers who had normal hearing and normal or corrected-to-normal vision. They attended school regularly and none of them had any history of neurological illness or brain damage. All the participants and their parents gave written informed consent to participate to the study. The study was approved by the local Ethics committee of the Université Grenoble-Alpes.

The children with dyslexia were recruited at the center for learning disabilities of the Grenoble University Hospital and in speech therapy offices. All participants had a normal IQ (exclusion if score score <25th percentile on the Raven’s Progressive Matrices (Raven et al., 1998) or if Verbal Comprehension Index and a Perceptual Organization Index lower than 85 on the Wechsler Intelligence Scale for Children – WISC IV; Wechsler, 2005). Children with dyslexia associated SLI or ADHD were not included. Reading age was evaluated with the Alouette Reading Test (Lefavrais, 1965). The dyslexic participants showed a reading delay of 38 months on average (mean reading age = 7 years and 5 months, *SD* = 8 months).

### Procedure and Timeline

Pretest measures included VA span, PA, phoneme categorical perception and reading measures. The tasks were proposed in a random order, except for the reading tasks that were always administered at the end of the assessment. Two software programs, MAEVA© and RapDys©, were then proposed to the participants for intensive training (6 weeks, 5 days a week, 15 min a day). Twenty-two children practiced first with RapDys©, 23 with MAEVA©. Post-test measures were collected after the first training session (Post-test1) and children were then engaged in a second training session using the other software, MAEVA© for the group previously trained with RapDys© and RapDys© for the group previously trained with MAEVA©. The participants’ skills were further assessed after the second training session (Post-test2).

### Assessment

#### Phoneme Awareness Tasks

The two PA tasks were the task of phoneme deletion from Bosse and Valdois (2009), and the acronyms task from the BELEC battery (Mousty et al., 1994). For each task, the participants were administered a set of practice trials for which they received feedback. No feedback was provided on the experimental trials. In the phoneme deletion task, the participants had to delete the first phoneme of a spoken word and pronounce the resulting pseudoword (e.g., “outil” /uti/→/ti/; “placard” /plakaR/→/lakaR/). Twenty experimental words were presented for which accuracy was recorded. Seven words began with a vocalic phoneme corresponding to a multiple letter grapheme so that the omission of the first letter (instead of the first phoneme) generated an incorrect response: nine began with a consonantal cluster, four with a singleton. In the acronyms task, participants were auditorily presented with pairs of words; they were instructed to extract the first phoneme of each word and blend them together to form a new syllable (e.g., “photo” “artistique” /foto/-/aRtistik/ says /fa/). The test comprised 10 trials of word pairs made up of 4.4 phonemes on average (range 2–8). Seven words began with a phoneme corresponding to a digraph so that children would produce an incorrect response if extracting the first letter instead of the first phoneme (response /pa/ instead of /fa/ if orthographically biased in the above example). A PA score was calculated as the average percentage of correct responses on the two phoneme deletion and acronyms tasks, performance was then computed as *Z*-scores by reference to normative data collected on typical readers (Zoubrinetzky et al., 2016).

#### VA Span Tasks

We administered global and partial letter report tasks to assess VA span abilities together with a task of single letter identification threshold to control for single letter processing speed. The tasks were displayed on a PC computer using E-prime software (E-prime Psychology Software Tools Inc., Pittsburgh, PA, United States). The strings were made of black upper case (Arial, 7 mm high) letters displayed on a white background at the center of the screen.

##### Global and partial report task

Five letter-strings (e.g., RHSDM; angular size = 5,4°) built up from 10 consonants (B, P, T, F, L, M, D, S, R, H) were displayed. The strings contained no repeated letters and did not match the skeleton of a real word (e.g., FLMBR for FLAMBER “burn”). Two subsequent letters never corresponded to a French grapheme (e.g., PH, TH) or a frequent bigram in French (e.g., TR, PL, BR). The distance between adjacent letters was of 0.57° in order to minimize crowding. In the Global Report condition, 20 five-letter strings were briefly displayed, centered on the fixation point. Each letter was presented 10 times, twice in each position. In the Partial Report condition, 50 random five-letter strings were used. Each letter occurred 25 times, five times in each position. For both tasks, a central fixation point was presented for 1,000 ms followed by a blank screen for 50 ms. A horizontal five-letter-string was then displayed for 200 ms, a duration which corresponds to the mean duration of fixations in reading, long enough for an extended glimpse, yet too short for a useful eye movement. In Global Report, children had to report all the letters they had identified immediately after the string offset. In Partial Report, a vertical bar cueing the position of the letter to be reported was displayed 1.1° below the target letter, at the offset of the letter-string. Each letter was a target once in each position. Participants had to report the cued letter only. In both tasks, the experimenter pressed a button to start the next trial after the participant’s oral response. The accuracy score corresponded to the number of accurately reported letters across the 20 trials in Global Report (regardless of order; maximum score = 100) or across the 50 trials in Partial Report (maximum score = 50). A VA span score was computed by averaging the percentage of correct responses on the two tasks of global and partial report. *Z*-scores were calculated from the normative data of Bosse and Valdois (2009).

##### Single letter identification task

To control for single letter identification skills, each of the 10 letters used in the report tasks were randomly displayed (five times each) at the center of the computer screen. The letters had the same physical characteristics as in the VA span tasks; they were presented for durations varying from 33 to 100 ms. A mask was displayed at the offset of the letter (13 mm high, 37 mm wide) for 150 ms. The child had to report the name of the letter immediately after its presentation. Ten practice trials preceded the test trials (two for each presentation time) for which participants received feedback. Participants for which the maximal score of 10 accurate identifications was not reached at least one of the presentation durations were not included in the sample. The total score was the sum of scores at each of the display durations.

#### Categorical Perception Tasks

A/dә/-/tә/ Voice Onset Time (VOT^2^) continuum, from −75 to +75 ms VOT with 30 ms steps, was synthetized by a parallel formant synthesizer provided by Carré (2004). VOT is negative when the onset of vocal vibrations begins before the burst of the plosive. VOT is positive when the onset of vocal vibrations begins after the plosive release. F1, F2, and F3 transition onset frequencies were 200, 2,200, and 3,100 Hz, respectively, and the steady-state formant parts were 500, 1,500, and 2,500 Hz, respectively. F0 frequency was maintained constant at 120 Hz. Each syllable of the continuum was 200 ms long. In previous studies using the same continuum, we checked that French-speaking typical adults (Hoonhorst et al., 2011) perceived negative VOT continuum endpoint as /dә/ and positive VOT continuum endpoint as /tә/.

We administered identification and discrimination tasks to the dyslexic participants. Two different cartoons from a children’s book (named Dom and Tom) were used to facilitate the association between sounds’ perception and the child’s response. Each cartoon was associated with a specific syllable (/dә/ or /tә/, respectively). The stimuli were binaurally delivered through headphones (Sennheiser HD 202).

##### Identification task

First, children completed a familiarization task composed of one block of 20 randomly presented stimuli (10 trials of each VOT endpoint values of the continuum: −75 ms and +75 ms VOT). The children had to associate each heard sound with the dedicated cartoon by pressing the keyboard “1” key if they heard the syllable /dә/ and the “0” key if they heard the syllable /tә/. The cartoons were displayed at the bottom of the screen; Dom was located on the left (above the “1” key) and Tom on the right (above the “0” key). Following each response, a feedback was provided on the screen (a red screen for incorrect responses, a picture of a gift for correct responses). The next trial was displayed 2,000 ms after the child response. At the end of the familiarization session, the experimental identification task was presented in one block of 60 randomly displayed stimuli (10 trials for each of the six VOT values: −75, −45, −15, +15, +45, and +75 ms VOT). No feedback was provided during the experimental task.

##### Discrimination task

The participants were first administered a familiarization task using the endpoints stimuli of the continuum. One block composed of randomly presented pairs of syllables (five trials of each of the following pairs: −75/−75 ms “/dә/-/dә/,” −75/+75 ms “/dә/-/tә/,” +75/−75 ms “/tә/-/dә/” and +75/+75 ms “/tә/-/tә/” VOT) was built up with a 100 ms interval between the pair’s stimuli. Two pairs of identical cartoons (Dom–Dom and Tom–Tom) were displayed on the right side of the screen close to the “0” response key that had to be pressed when the two syllables were phonologically the same, i.e., for either the /dә/-/dә/ or /tә/-/tә/ pairs. The participants were asked to press the “1” key (on the left side close to the two pairs of different cartoons, i.e., Dom–Tom and Tom–Dom) when the syllables were different, whatever their order (/dә/-/tә/ or /tә/-/dә/). A feedback was provided on the screen after the response (a red screen or a gift picture) and the next trial was presented 2,000 ms after the child response. The discrimination task was subsequently presented to each child, composed of a set of 80 pairs of stimuli, displayed in a random order (five trials of each of the eight same pairs: −75/−75, −45/−45, −15/−15, +15/+15, +45/+45, and +75/+75 ms VOT; and five trials of each of the 10 different pairs: −75/−45, −45/−75, −45/−15, −15/−45, −15/+15, +15/−15, +15/+45, +45/+15, +45/+75, and +75/+45 ms VOT). Neither positive nor negative feedback was provided during the task.

##### Phonemic discrimination score

The discrimination data were used to calculate five discrimination scores, each corresponding to the mean of the responses to same and different stimulus pairs centered on −60, −30, 0, +30, and +60 ms (e.g., for the 0 ms VOT pair: mean of the response’s to the −15/−15, +15/+15, −15/+15, +15/−15 pairs). For each stimulus pair, the discrimination scores were converted into *d*-prime (*d*’) scores by taking the difference between the standard normal deviates (*Z*-values) of the same and different pairs (Mcmillan et al., 1977). A phonemic discrimination score was then calculated by taking the difference between the 0 ms VOT discrimination d’ score and the mean of the four other *d*’ scores (−60, −30, +30, and +60 ms VOT).

#### Reading Tasks

To assess the evolution of reading skills while avoiding potential test–retest effects, reading tasks of regular words, irregular words and pseudowords and a text reading task were created in three versions (A, B, and C), for each of the three assessment sessions (Pre-test, Post-test1, and Post-test2). We administered these four tasks in a random order, but always at the end of the cognitive assessment.

The children had to read aloud 27 regular words, 27 irregular words, and 27 pseudowords matched in length. Specifically, the pseudowords were created by substituting a few letters of the regular words, while keeping their graphemic and syllabic structure similar (e.g., ‘capsule’ becomes ‘copsale’ or ‘tente’ becomes ‘taude’). The words used in each of the three A, B, and C versions were matched in lexical frequency, from the MANULEX database (Lété et al., 2004). The words and pseudowords were presented in columns in black lower case (Times, 14) on a white A4 sheet. The children had to read the items from top to bottom, as quickly and as accurately as possible. They were informed of the nature of the items (words or pseudowords) prior to reading. They were further asked to read a short text aloud (mean words number = 140 words) during 1 min. The A, B, and C texts were matched for word frequency and grammatical structure and contained the same sentence number and the same number of words of different grammatical categories (from the test battery Diagnos: Denhière et al., 1991). Reading time and reading errors were recorded for each task. The pseudoword, regular word, irregular word and text reading scores corresponded to the number of words correctly read per minute.

### Training Groups

The children were trained with one of the two programs during the first session and then switched to the other program for the second session. Half of them (*N* = 22) practiced first with MAEVA© (MAEVA©–RapDys© order, hereafter M–R order), the other half (*N* = 23) started with RapDys© (RapDys©–MAEVA© order, hereafter R–M order). As shown on Table 1, the two training groups were matched in chronological age, reading age, phonemic perception performance, VA span, and reading skills but they differed in PA skills. Despite random assignment of the participants to each group, the group that started with the RapDys© program had lower PA skills.

**TABLE 1:** Characteristics of the two training groups.

	**MAEVA©/RapDys© (*N* = 22)**	**RapDys©/MAEVA© (*N* = 23)**	**Difference**	**Whole sample (*N* = 45)**
				
	**Mean (SD)**	**Mean (SD)**	**F tests**	**Mean (SD)**
Age (months)	127 (17)	126 (16)	*F* < 1	126 (16)
Reading age (months)	89 (7)	88 (10)	*F* < 1	88 (8)
Reading delay (months)	−38 (15)	−38 (13)	*F* < 1	−38 (14)
Phonemic perception (*d*’ score at 0 ms VOT)	1.4 (1.1)	1.6 (1.1)	*F* < 1	1.5 (1.1)
Visual attention span				
Raw score	73 (11)	72 (14)	*F* < 1	72 (12)
*Z*-score	−1.17 (1.0)	−1.16 (1.1)	*F* < 1	−1.16 (1.05)
Phonemic awareness				
Raw score	75 (15)	69 (18)	*p* = 0.245	71 (16)
*Z*-score	−0.50 (0.9)	−0.79 (1.0)	*p* = 0.245	−0.65 (0.9)
Pseudo-word reading (wpm)	17 (8)	17 (9)	*F* < 1	17 (8)
Regular word reading (wpm)	30 (16)	30 (19)	*F* < 1	30 (18)
Irregular word reading (wpm)	16 (11)	16 (15)	*F* < 1	16 (13)
Text reading (wpm)	64 (31)	66 (37)	*F* < 1	65 (34)

### Training Programs

#### MAEVA©

The MAEVA© software was developed to improve VA span, i.e., to increase the number of visual items to be processed simultaneously at a glance. Training was based on visual categorization tasks involving five categories (or families) of characters: lowercase letters, pseudo-letters, numbers, Japanese Hiragana characters, unknown geometric shapes.

Using the algorithm proposed by Pelli et al. (2006), we ensured that visual identification efficiency was similar between categories. The size of each character was fixed at 1° when perceived 60 cm from the screen. The characters were presented in black on a white screen. Between-characters spacing was enlarged (1.3 times normal spacing) to minimize crowding effects.

Each training session began by a 5-min familiarization to the different character families. Each familiarization trial began with the display of a fixation cross at the center of the screen, followed by the presentation of one character for 100 ms, immediately followed by a mask for 500 ms. A response screen with the five labels representing the family’s characters was then presented, and the child had to click on the label of the family corresponding to the perceived character, then on a central arrow to start the next trial.

During the training program, the participants were engaged in a categorization task similar to that used in a previous study (Lobier et al., 2014). Three parameters were manipulated across trials: the number of characters within string, presentation duration and task difficulty. The number of characters within a string varied from two to seven, and presentation time from 420 to 120 ms. The tasks varied in difficulty from the easier (1) to the more difficult (6) depending on the instructions: (1) How many families have you seen? (2) Did you see elements of this family? (3) Which families were present? (4) How many elements of this family did you see? (5) How many each family members were there? (6) Which families were present and how many elements of each family was there?

To improve children’s VA span and keep their attention and motivation sustained, we used an adaptive algorithm adapted from Wilson et al. (2006). The algorithm adapted the difficulty of each trial online, depending on the child previous responses. Based on previous trials, the algorithm calculated the probability of each potential trial (defined by X number of items, T presentation time, and Z instruction) to be successfully managed. The next proposed trial was chosen to have an estimated success rate around 75%, which was expected to be the best condition to optimize learning. The algorithm ensured a progression from longer to shorter durations, from shorter to longer character strings and from the easier to the more difficult tasks.

**FIGURE 1:**
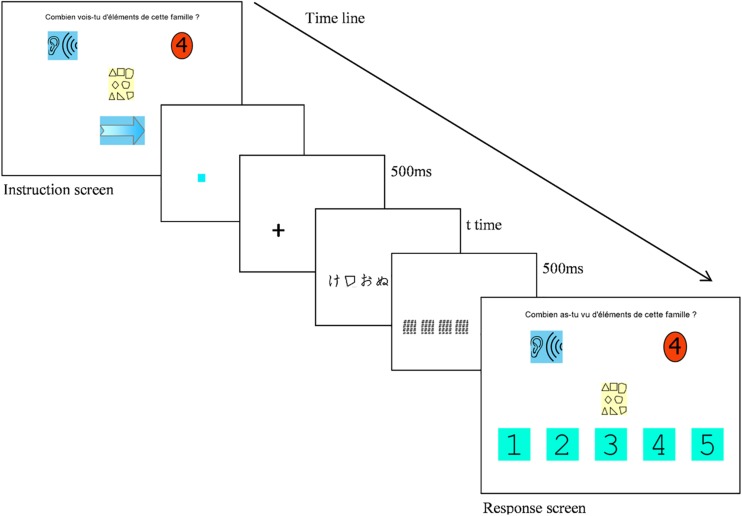
Example of a trial for MAEVA© training. Instruction n° 4 (How many elements of this family did you see?) is presented; T time is the presentation time defined by the algorithm.

As illustrated on Figure 1, at the beginning of each trial the participant clicked on a little blue square that was randomly displayed anywhere on the screen. At the click, a fixation cross was displayed for 500 ms in place of the blue square. Then, the character string was presented for a duration defined by the algorithm. Random stimulus display position on the screen was required to maintain a high level of attention all along the session, and optimize perceptual learning (Harris et al., 2012). A mask made up of random lines of the same width as the stimuli was displayed for 500 ms at the offset of the string. A response screen was then presented including the instructions and the label(s) together with the button responses on which the child had to click (see 1 for an illustration). Feedback was given in a written form at the top of the screen (BRAVO or DOMMAGE), accompanied by a symbolic feedback (a happy or sad emoticon). A score counter was presented at the bottom right of the screen to maintain motivation. The child clicked the blue arrow to move to the next trial.

#### RapDys©

**FIGURE 2:**
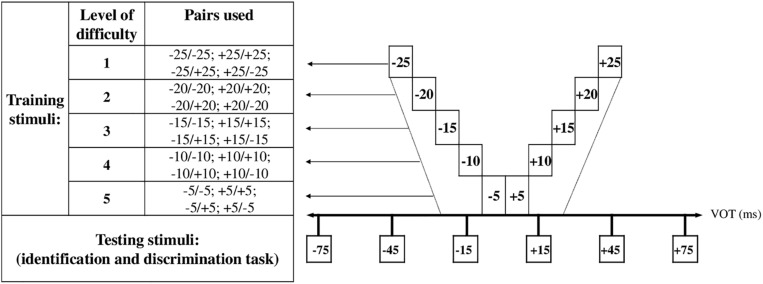
VOT values used in RapDys© for the identification and discrimination tasks (from Collet et al., 2012, Figure 1).

The RapDys© software (running under MATLAB Compiler Runtime V.8.1.) used in this study is an adaptation of the software used by Collet et al. (2012). The method is based on the perceptual fading task (Jamieson and Morosan, 1989). Basically, the idea was to gradually decrease the acoustic distance between two phonemes presented in a task discrimination. The pairs were synthesized to create a /dә/-/tә/ continuum varying in VOT. The gradual reduction of the acoustic distance was set up within five levels of increasing difficulty tailored to the child’s performance. Each training session consisted of five blocks of 20 trials. Figure 2 depicts the different levels of difficulty and their associated VOT values. The sessions always started by the easier pair condition (level 1) and difficulty increased when the child reached a 80% (16/20) correct success rate on one block. The session ended after the award of five blocks, regardless of the level reached. However, to adjust the duration of each session at around 15 min depending on the child’s performance and prevent best-performing children from fast execution strategies, especially after several weeks of training, a sixth block was added (bonus block). This block was composed of the same stimuli as the fifth block. It was only proposed to the children who got an above 80% success rate on all five blocks. The pairs were presented in a random order that differed for each child and each session.

Each phoneme was associated with the same cartoon as previously used for assessment. At the beginning of each training session block, a soundtrack introduced the child to the task. The cartoons were displayed on the computer screen all along the task: the two pairs of identical cartoons in the lower right corner of the screen, near to the key 0 response button and the two pairs of different cartoons (one for each order) at the bottom left corner near key 1. A present was displayed centered at the top of the screen. When the child heard the same sound twice (e.g., /dә/-/dә/ or /tә/-/tә/) he/she was instructed to press key 0, below the Dom–Dom and Tom–Tom cartoons; when he/she heard two different sounds (e.g., /dә/-/tә/ or /tә/-/dә/), the instruction was to press key 1, below the Dom–Tom/Tom–Dom cartoons. When the response was correct, the present moved from the top to the corresponding cartoons and stayed displayed above them. If the answer was wrong, the present was crossed. Feedback on the response disappeared after 2,000 ms and the next trial began. At the end of each block of 20 trials, the score (% of correct answers) and a short message was displayed on the screen to encourage the child: ‘Courage! Hold on!’ when success rate was less than 50% or ‘Congratulations! Keep trying like this!’ when success rate was higher than 50%. The instruction was repeated at the beginning of each new block. It was suggested to the child to track his progress after each block and each session in order to promote motivation and improve performance at the next session.

### Analysis Strategy

As we used a cross-over design to compare the effects of the two methods, without untrained control group, a difference between the effects of the methods was needed to exclude a mere test–retest effect. We used the following analysis strategy in order to test the difference between methods in the most powerful conditions. All the tests were performed with the SPSS 24© software.

Two different contrasts were used to assess the overall effect, together for the two presentation orders, of each training method (RapDys© and MAEVA©) on the different variables under scope (phonemic perception, VA span, PA, and pseudoword, regular word, irregular word and text reading). The first of these contrasts [C1] compared the Post-test1 (T1) score to the Pre-test score (T0). The second contrast [C2] compared the Post-test2 (T2) score to the mean of the T1 and T0 scores.

[C1]: T1 score – T0 score
[C2]: T2 score – (T0 score, T1 score)/2
  • Orthogonality condition: sum of cross-products of C1 and C2 coefficients = 0.

C1 and C2 are ‘orthogonal’ contrasts (Hays, 1988): they allow independent assessment and testing of the effects of the first and second training, i.e., training estimations are not correlated (over participants, over studies with different participants). With non-orthogonal contrasts (here: T1 score – T0 score; T2 score – T1 score), the assessment of the two trainings would be inversely correlated, i.e., when the effect one of the trainings is overestimated, the effect of the other training is underestimated (see Supplementary Material). Using orthogonal contrasts thus avoids to cumulate errors in the estimations of the first and second trainings. A further reason for using the mean of T0 and T1 scores as baseline for estimating the effects of the second training is that individual differences in performances at T2 not only depend on those at T1 but also on those at T0 (see Revision Comments). The mean of T1 and T0 scores is thus a more reliable baseline than T1 alone for assessing the effect of the second training.

C1 and C2 corresponded to one of the two methods for a given order and they were assigned to a different level of the Method factor depending on the Order. One of the two levels of the Method factor corresponded to the RapDys© effect and it was calculated either as C1, for the group that was given the RapDys© training in first instance (R–M order), or as C2, for the group that was given the RapDys© training in second instance (R–M order). The alternative level of the Method factor corresponded to the MAEVA© effect and it was calculated either as C1, for the group that was given the MAEVA© training in first instance (M–R order), or as C2, for the group that was given the MAEVA© training in second instance (R–M order).

The two orthogonal contrasts were entered in a repeated-measures Method (MAEVA©, RapDys©) × Order (M–R, R–M) ANOVA as two independent levels of the Method factor. The advantage of this ANOVA design is that the Method effect is tested on the combined the data of the two subgroups (for a similar design see Methods-fMRI statistics in Brem et al., 2010). With a Time × Order ANOVA, the effect of the Method would be tested as a difference between subgroups, a procedure that is less powerful.

If the Method effect was not significant and there was a trend toward significance, conclusions were based on separate assessments of the effect of each method with *t*-tests. If the Method effect was not significant and if there was at least a trend toward significance for the Method × Order interaction, the Method effect was tested separately for each Order. We concluded in favor of a difference between methods if there was a significant difference in favor of one method for at least one of the two orders.

## Results

### Phonemic Perception

**FIGURE 3:**
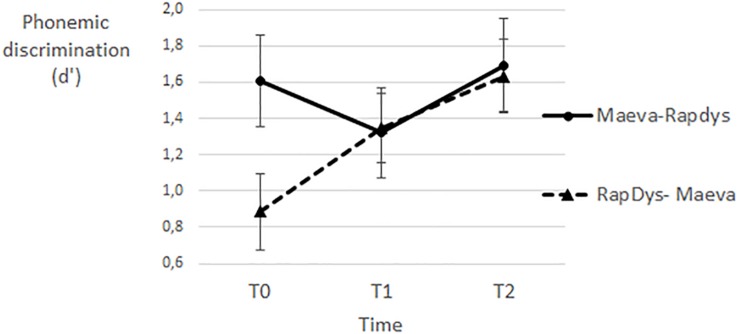
Phonemic discrimination peaks (at 0 ms along the VOT continuum; in *d*’ values) for each time period (pre-training, post-training 1; post-training 2) and each presentation order (left: R–M, right: M–R).

Figure 3 gives the magnitudes of the phonemic discrimination peaks for each order and each of the three time periods (T0, T1, T2). For the M–R order, the phonemic peak decreased at T1 but increased at T2. For the R–M order, there were fairly similar increases in the magnitude of the phonemic peak at T1 and at T2, and the overall increment was better than for the M–R order.

A repeated-measures Method (MAEVA©, RapDys©) × Order (M–R, R–M) ANOVA was performed with the phonemic discrimination score as dependent variable. The Method effect was not significant [*F*(1,43) = 1.61, *p* = 0.21, η^2^ = 0.036]. The Order effect was significant [*F*(1,43) = 6.23, *p* < 0.05, η^2^ = 0.127], indicating a larger improvement, irrespective of the Method, for the R–M order compared to the M–R one (Figure 3).

The Method × Order interaction was not significant but there was a trend toward significance [*F*(1,43) = 2.35, *p* = 0.13, η^2^ = 0.052]. When tested separately per order, the Method effect was not significant for the R–M order (*F* < 1) and marginally significant for the M–R order [*F*(1,21) = 3.55, *p* = 0.07, η^2^ = 0.145]. The absence of Method effect when RapDys© is presented in first instance (R–M order) can be attributed to a long term effect of RapDys©. Such enduring effect might also explain the better overall improvement for the R–M order compared to the M–R one (the significant Order effect).

### Visual Attention Span

**FIGURE 4:**
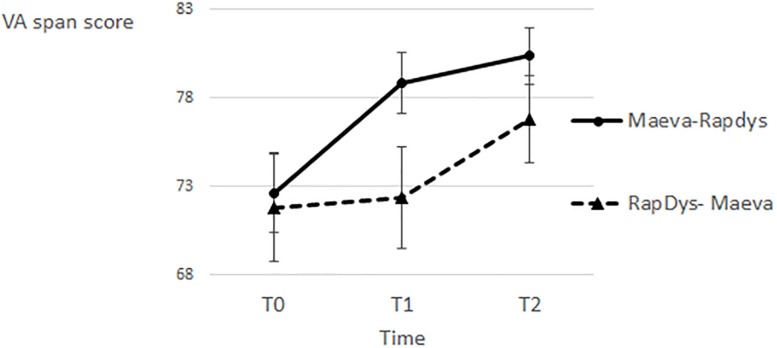
Visual attention span scores for each time period and each presentation order.

For both presentation orders, there was a fairly larger increase in VA span after MAEVA© training and a much smaller increase in VA span after RapDys© training (Figure 4). The overall increment was better for the M–R order than for the R–M order.

A repeated-measures Method (MAEVA©, RapDys©) × Order (M–R, R–M) ANOVA was performed, with the VA span score as dependent variable. The Method effect was significant [*F*(1,43) = 5.49, *p* < 0.05; η^2^ = 0.113], showing that MAEVA© was more relevant overall than RapDys© to improve VA span. The Order effect was also significant [*F*(1,43) = 4.44, *p* < 0.05, η^2^ = 0.094], indicating a larger improvement, irrespective of the Method, for the M–R order compared to the R–M one (Figure 4).

### Phonemic Awareness

**FIGURE 5:**
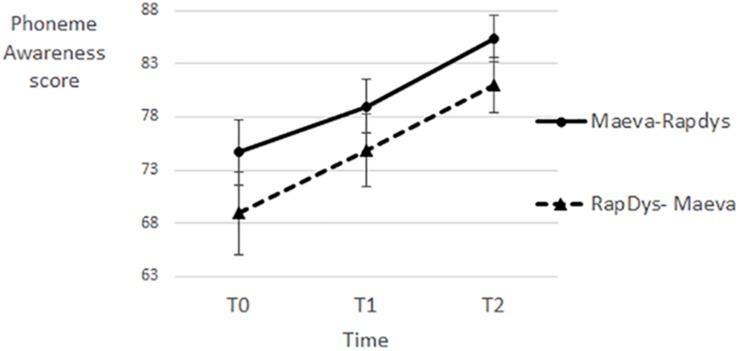
Phonemic awareness scores for each time period and each presentation order.

As shown on Figure 5, the effects of the two training methods on PA are fairly similar, for both presentation orders. A repeated-measures Method (MAEVA©, RapDys©) × Order (M–R, R–M) ANOVA was performed, with the PA score as dependent variable. The Method and Order effects were not significant (both *F* < 1) and the Method × Order interaction was marginally significant [*F*(1,43) = 3.37, *p* = 0.07, η^2^ = 0.073]. When tested separately per order, the Method effect was not significant for either the R–M order [*F*(1,22) = 2.56, *p* = 0.12, η^2^ = 0.104] or the M–R order [*F*(1,21) = 1.42, *p* = 0.25, η^2^ = 0.063]. The results of these univariate ANOVAs suggest that there are no significant differences between the effects of the methods, despite the presence of a nearly significant Method × Order interaction. Further, when tested separately per method with *t*-tests, both the RapDys© effect and the MAEVA© effect were significant [*t*(44) = 5.66, *p* < 0.001, η^2^ = 0.421; 3.48, *p* = 0.001, η^2^ = 0.216, respectively]. Thus, PA improved in much the same way following either RapDys© or MAEVA© training.

### Pseudo-Word Reading

**FIGURE 6:**
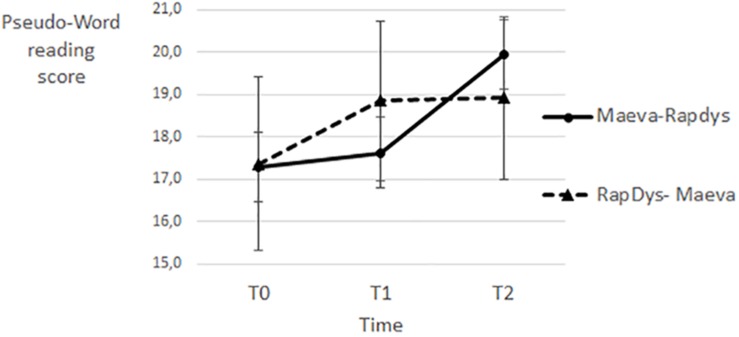
Pseudo-word word reading scores for each time period and each presentation order.

As shown in Figure 6, pseudo-word reading performance improves more drastically following RapDys© than MAEVA© training for the two presentation orders. A repeated-measures Method (MAEVA©, RapDys©) × Order (M–R, R–M) ANOVA was performed, with the pseudo-word reading score as dependent variable. The Method effect was not significant but there was a trend [*F*(1,43) = 2.17, *p* = 0.15, η^2^ = 0.048], and both the Order effect and the Method × Order interaction were not significant (both *F* < 1). When tested separately per method with *t*-tests, the RapDys© effect was significant [*t*(44) = 2.57, *p* < 0.05, η^2^ = 0.131], but the MAEVA© effect was not significant (t<1). The overall results suggest that RapDys© is more relevant than MAEVA© to improve pseudo-word reading.

### Regular Word Reading

**FIGURE 7:**
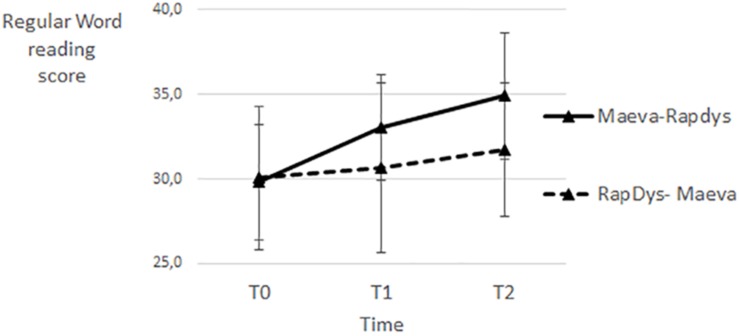
Regular word reading scores for each time period and each presentation order.

The effects of the two training methods on regular-word reading are illustrated on Figure 7. Both methods have fairly similar effects. However, the improvements are larger for the M–R order than for the R–M order.

A repeated-measures Method (MAEVA©, RapDys©) × Order (M–R, R–M) ANOVA, with the regular word reading score as dependent variable, revealed that the Method effect, and the Method × Order interaction were not significant (both *F* < 1). The Order effect was not significant [*F*(1,43) = 2.60, *p* = 0.11, η^2^ = 0.057]. When tested separately per method, the RapDys© effect was significant [*F*(1,44) = 4.59, *p* < 0.05, η^2^ = 0.094], and the MAEVA© effect was not significant although there was a trend [*F*(1,44) = 2.31, *p* = 0.13, η^2^ = 0.050]. The lack of significance of the MAEVA© effect, although the mean effect of this method was larger than the one of RapDys© (Figure 7), is due to a higher variability. The overall results suggest that the two methods have similar effects on regular word reading.

### Irregular Word Reading

**FIGURE 8:**
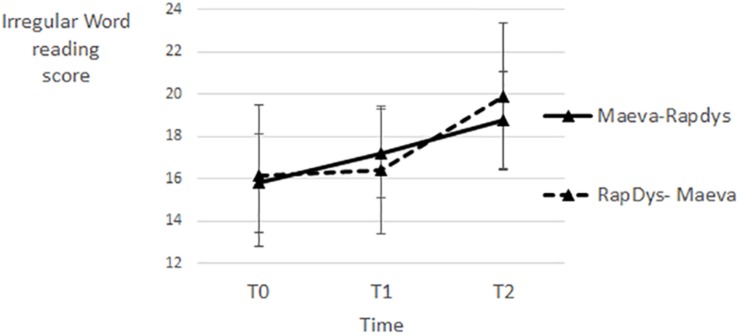
Irregular word reading scores for each time period and each presentation order.

The effect of MAEVA© on irregular word reading seems larger than the effect of RapDys© for both presentation orders (Figure 8). A repeated-measures Method (MAEVA©, RapDys©) × Order (M–R, R–M) ANOVA was performed, with the irregular word reading score as dependent variable. The Method and Order effects were not significant [*F*(1,43) = 1.73, *p* = 0.19, η^2^ = 0.039; *F* < 1, respectively], but the Method × Order interaction was significant [*F*(1,43) = 5.18, *p* < 0.05, η^2^ = 0.107]. When tested separately per Order, the Method effect was significant for the R–M order [*F*(1,22) = 7.31, *p* < 0.05, η^2^ = 0.250], and not significant for the M–R order (*F* < 1). The overall results suggest a positive and higher effect of MAEVA© on irregular word reading. The absence of Method effect when MAEVA© is presented in first instance (M–R order) may be due to a long-term effect of MAEVA©.

### Text Reading

**FIGURE 9:**
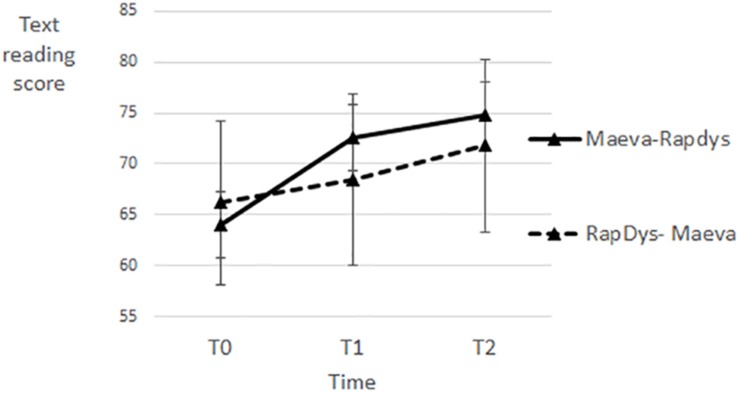
Text reading scores for each time period and each presentation order.

The effects of RapDys© and MAEVA© on text reading are illustrated on Figure 9. A repeated measures Method (MAEVA©, RapDys©) × Order (M–R, R–M) ANOVA was performed, with text reading score as dependent variable. The Method effect and the Method × Order interaction were not significant (both *F* < 1). The Order effect was not significant [*F*(1,43) = 1.61, *p* = 0.21, η^2^ = 0.036]. When tested separately per method, both the RapDys© and MAEVA© effect were significant [*t*(44) = 2.12, *p* < 0.05, η^2^ = 0.093; 2.62, *p* < 0.05, η^2^ = 0.135], suggesting that the two methods have similar positive effects on text reading.

### Summary of the Results

The results of the statistical tests are summarized in Table 2. Only the tests that were used to reach a conclusion are reported. The Method effect was only significant for VA span (*p* < 0.05), supporting a stronger effect of MAEVA compared to the one of RapDys irrespective of the presentation order. For pseudo-word reading, the Method effect was not significant (*p* = 0.15). However, separate tests per method evidenced a significant effect of RapDys and no significant effect of MAEVA. For three other variables (phonemic perception, PA, and irregular word reading), the Method × Order interaction was either significant or there was a trend toward significance. For these variables, the Method effect was tested separately for each order. For phonemic perception, we concluded in favor of a larger effect of RapDys, compared to the one of MAEVA, because the improvement in phonemic discrimination was larger (*p* < 0.07 trend toward significance) with RapDys than with MAEVA when the presentation order excluded the persistence of a previous effect of RapDys (M–R order). Similarly, for irregular word reading, we concluded in favor of a larger effect of MAEVA, compared to the one of RapDys because the improvement was larger (*p* < 0.05) with MAEVA than with RapDys when the presentation order excluded the persistence of a previous effect of MAEVA (R–M order). Finally, for PA, the difference between methods were not significant for both orders, suggesting that both methods had fairly equivalent effects.

**TABLE 2:** Summary of the statistical tests (R = RapDys; M = MAEVA).

	**Phonemic perception**	**VA span**	**Phonemic awareness**	**Pseudo-word reading**	**Regular word reading**	**Irregular word reading**	**Text reading**
Method effect	*p* = 0.21	*p* < 0.05	*F* < 1	*p* = 0.15	*F* < 1	*p* = 0.19	*F* < 1
Method × Order Interaction	*p* = 0.13	*p* = 0.30	*p* = 0.07	*F* < 1	*F* < 1	*p* < 0.05	*F* < 1
Method effect per Order	R–M: *F* < 1		R–M: *p* = 0.12			R–M: *p* < 05	
	M–R: *p* = 0.07		M–R: *p* = 0.25			M–R: *F* < 1	
Separate test per Method				R: *p* < 05			
				M: *t* < 1			
CONCLUSION	RapDys > MAEVA	MAEVA > RapDys	No difference	RapDys > MAEVA	No difference	MAEVA > RapDys	No difference

Table 3 presents the changes expressed in effect sizes (Cohen’s *d*; Cohen, 1988) for each variable of interest and each training method, irrespective of the presentation order. The mean values of the training effects were tested with *t*-tests performed on the whole sample (and training effects assessed with C1 and C2 contrasts, depending on whether the training was used in first or in send instance, respectively). Shaded areas indicate probable long-lasting (indirect) effects of the alternative method. RapDys© had a medium effect on phonemic perception and pseudo-word reading and it had a very large effect on phoneme awareness. MAEVA© had a very large effect on VA span and a rather large effect on irregular word reading and PA. The training effects on word and text reading did not depend on the method and were fairly small.

**TABLE 3:** Summary of the effect sizes (Cohen’s *d*; ^∗∗∗^*p* < 0.001; ^∗∗^*p* < 0.01; ^*^p < 0.05).

	**Phonemic perception**	**Visual attention span**	**Phonemic awareness**	**Pseudo-word reading**	**Regular word reading**	**Irregular word reading**	**Text reading**
RapDys©	0.47^*^	0.63^∗∗^	1.21^∗∗∗^	0.55^*^	0.46^*^	0.43^*^	0.45^*^
MAEVA©	0.35	1.19^∗∗∗^	0.74^∗∗^	0.18	0.32	0.76^∗∗∗^	0.56^*^

*Shaded ares indicate probable lasting effects of the alternative method.*

## Discussion

The purpose of the current study was to explore the effect of two training methods, RapDys© and MAEVA©, designed to improve phoneme categorical perception and VA span, respectively, in dyslexic children. A first important step was to verify the efficacy of the two softwares, i.e., their ability to improve the cognitive mechanisms they were supposed to train. A second main concern was whether categorical perception training resulted in better phoneme awareness. A last key point concerns the ability of the two methods to improve reading performance.

### Efficacy of RapDys© and MAEVA© Training

Results clearly show that MAEVA© more than RapDys© improves VA span and that the amplitude of the effect is very large, suggesting that MAEVA© is relevant to train VA span. The effect of MAEVA© on VA span is not trivial as the tasks and material used for training differed from those used for pre–post training assessment. With MAEVA©, children were trained on non-verbal categorization tasks, using mainly non-literal material (letters, pseudo-letters, digits, geometrical figures, Hiragana characters) while pre–post intervention VA span was assessed through the oral naming of consonant strings. It follows that the capacity of MAEVA© to improve VA span cannot be attributed to higher letter familiarity or a task effect. This is quite in line with previous evidence that VA span is not sensitive to the type of material, verbal or non-verbal, to be processed (Lobier et al., 2012b). Independently of the type of stimuli, all training trials required rapid multi-element processing in conditions of presentation time that favored parallel processing. Thus, the efficacy of MAEVA training on VA span more likely reflects the ability to process longer multi-character strings faster thanks to increased VA capacity (Lobier et al., 2013). In support of this interpretation, neuroimaging data revealed that VA span relates to the brain superior parietal regions involved in VA (Peyrin et al., 2012; Reilhac et al., 2013) and that these brain regions respond similarly to verbal and non-verbal elements (Lobier et al., 2012a, 2014). Otherwise, the large size effect of MAEVA© training on VA span (Cohen’s *d* ≈ 1.20) demonstrates that MAEVA© was well designed to target VA and improve parallel multi-character processing. A key finding of the current study is thus to demonstrate that MAEVA© is efficient to improve VA span. The inclusion of an adaptive algorithm that adjusts the exercises online to each child needs probably contributes to explain the large effect size observed on VA span tasks following MAEVA© training.

The effect of RapDys© on phonemic perception, compared to the one of MAEVA©, was modulated by training order. When RapDys was presented in second instance, its effect was stronger than the one of MAEVA. When RapDys was presented in first instance, its effect was comparable to the one of MAEVA©, probably due to long-lasting effect of previous practice with RapDys©. The effect of RapDys© on phonemic discrimination was relatively small. This is in accordance with previous findings reported in SLI and mainstream children (application to RapDys© to children with SLI: Collet et al., 2012; application of a similar procedure to mainstream children: Moore et al., 2005). One possible explanation is that the stimulus pairs at the end of the discrimination training (VOT contrasts narrowing from −25/+25 to −5/+5 ms) were closer to the phoneme boundary than those used for assessing the training effects (−15/−15 ms VOT contrast). As discrimination training in some stimulus region (e.g., around the phonemic boundary) shifts neural resources toward this region (Guenther et al., 2004), then stimuli that fall at its margins might not entirely benefit from training.

### Effect of RapDys© on Phoneme Awareness

Assuming that categorical perception (Manis et al., 1997) but not VA span (Zoubrinetzky et al., 2016) modulates PA, practicing RapDys© was expected to improve PA more efficiently than practicing MAEVA© in our dyslexic population. However, while training with RapDys© had a strong positive effect on PA, PA improvement was also observed following MAEVA© and there was no significant difference between the effects of the two methods.

Such effect of MAEVA on PA through VA span improvement was rather unlikely. Previous studies have shown the absence of significant link between VA span and PA (after control of age and IQ) in both typical readers and children with dyslexia (Bosse et al., 2007; Bosse and Valdois, 2009; Zoubrinetzky et al., 2016; Banfi et al., 2018; Zhao et al., 2018). Moreover, an effect of VA span on PA would predict systematic PA deficit in children with impaired VA span, against behavioral evidence that the two deficits typically dissociate in the dyslexic population (e.g., Bosse et al., 2007; Zoubrinetzky et al., 2014; see however, Saksida et al., 2016). Neuroimaging data showing that a selective VA span deficit alters normal activation of the superior parietal lobules bilaterally without concomitant effect on the brain areas involved in PA is further evidence against a direct link between VA span and PA (Peyrin et al., 2012). Nevertheless, the improvement in phoneme awareness after MAEVA training in the present study suggests that MAEVA training might have implications for PA. As MAEVA training is expected to improve multi-letter orthographic units parallel processing, better grapheme processing might have facilitated phoneme awareness (Vidyasagar and Pammer, 2010). However, effects of concomitant phonic training cannot be ruled out (as there was no control group).

In contrast, there is previous report of positive effects of RapDys© on PA in SLI children. Collet et al. (2012) showed greater PA improvement in a group of SLI children who benefited from intensive RapDys© training than in a control group who benefited from no specific training. The current results suggest that the RapDys© effect is not limited to children with specific language impairment but further extends to children with dyslexia (but no SLI), thus suggesting that RapDys© has the potential to improve PA when impaired, whatever the child neurodevelopmental disorder. The existence of a link between categorical perception and PA (Manis et al., 1997; Zoubrinetzky et al., 2016) and evidence that PA improves following phoneme discrimination training in mainstream school children (Moore et al., 2005), SLI children (Collet et al., 2012) and dyslexic children (the current study) support a causal relationship.

Another main finding of the current paper is that the improvement in PA following phoneme discrimination training is quite substantial. The effect size of PA improvement following RapDys© discrimination training (Cohen’s *d* ≈ 1.20) is larger than previously reported for the remediation methods that explicitly targeted PA. In their meta-analysis of intervention studies on typically developing US children, Bus and van IJzendoorn (1999) reported a medium-to-strong effect (*d* = 0.73) of PA training on PA performance. The impact of PA training on PA skills was also found significant in the US National Reading Panel’s meta-analysis (Ehri et al., 2001) but again the effect size (*d* = 0.86) was smaller than in the current study. As typically developing children were found to profit more from PA training than disabled readers in this later study, the impact of RapDys© on PA skills is very impressive. It is further noteworthy that such significant effects were obtained after only a few sessions corresponding to only 7 h and 30 min of training.

### Complementary Effects of RapDys© and MAEVA© on Reading Subskills

Since both RapDys© and MAEVA© successfully improve reading-related cognitive skills, PA and VA span, respectively, both were expected to have positive impact on reading. We here more specifically explored whether each training had specific and complementary effects on different reading subskills. RapDys© more specifically improves pseudo-word reading while MAEVA© more specifically improves irregular word reading. In contrast, there was no differential effect of the methods on regular word and text reading.

It is widely accepted that the ability to isolate and manipulate phonemes in spoken words is critical to develop decoding skills (Ehri et al., 2001) and that PA more specifically relates to pseudo-word reading (Sprenger-Charolles et al., 2006). Pre-reading PA is predictive of later pseudo-word reading in typical children (Shapiro et al., 2013), higher PA sensitivity is associated with better pseudo-word reading in bilingual children (Bialystok et al., 2005) and a PA deficit can result in selectively impaired pseudo-word reading in developmental dyslexia (Peterson et al., 2013). There is thus ample evidence for a favored relationship between PA and pseudo-word reading, suggesting that the positive effect of RapDys© on pseudo-word reading might follow from its capacity to improve PA. An indirect effect path from speech perception training to reading would be in agreement with evidence from a developmental study that the association between phoneme perception and reading is indeed mediated by PA (McBride-Chang et al., 1997). However, a direct path from speech perception to reading was also found in a subsequent study (Zhang and McBride-Chang, 2014). Thus, a direct effect of training phoneme categorical perception on reading cannot be excluded.

Many studies on typical readers and children with dyslexia have explored the relationship between VA span and reading (Bosse et al., 2007; Lobier et al., 2013; van den Boer et al., 2013; Zoubrinetzky et al., 2016). Results typically show that VA span contributes to both pseudo-word and irregular word reading but a more sustained relationship with irregular word reading has been reported in typical readers (Bosse and Valdois, 2009) and a selective irregular word reading deficit is associated with the VA span deficit in some dyslexic children (Valdois et al., 2003; Dubois et al., 2010). Simulations within the framework of the multi-trace memory model of reading (Ans et al., 1998) also comfort the link between VA span and irregular word reading, showing that VA span damage primarily disturbs irregular word reading. There is a general consensus that irregular word reading does not rely on decoding skills but on whole-word recognition by sight (Coltheart et al., 2001). Assuming that whole-word recognition implies the simultaneous processing of all the word letters and that improving VA span allows processing more letters simultaneously, it naturally follows that VA span improvement through MAEVA© training would result in better irregular word reading, as observed.

Finally, the overall effects of RapDys© and MAEVA© on reading performance are quite noteworthy, all the more when one considers that training was only administered for a short duration of 7 h and a half and that participants were very severely impaired children who showed a 38 months delay in reading, thus having a Grade 1 reading level before intervention. Typically, positive effects of PA training on reading performance are reported after longer training durations in populations of poor readers (Torgesen et al., 2001) and children with the most severe reading impairment show very poor growth in reading, if any, following intensive PA training (Ehri et al., 2001). The current findings thus suggest that targeting categorical perception as a precursor of PA is very promising to improve reading skills in severely impaired children with dyslexia who are resistant to most current trainings. In the same way, the selective and large effect of VA span training on irregular word reading is further evidence that VA span is critical for whole-word processing, so that selective VA-span training may further improve orthographic knowledge acquisition and spelling performance (Bosse et al., 2015; van den Boer et al., 2015).

## Conclusion

In conclusion, the current study provides evidence of the efficacy of RapDys© and MAEVA© training to improve categorical perception and VA span, respectively. Categorical perception training further appears critical to improve PA and pseudoword reading while VA span training is critical to improve irregular word reading and it might also contribute to improve PA. That each training program selectively affects a different reading subskill supports the independence of the PA and VA span deficit in developmental dyslexia. For the first time, practitioners would have two complementary training programs at their disposal to substantially help children with a double PA and VA span deficit. While PA may indirectly contribute to irregular word reading and VA span to pseudo-word reading (Zoubrinetzky et al., 2014), the direct impact of each program to one of these cognitive skills would more efficiently improve reading performance in double-deficit children when combined.

Future studies are needed to explore how to adjust the duration of trainings to each child specific needs. It is well admitted that improvement in reading outcomes partly depends on training duration and we expect more severely impaired children with dyslexia to require more training to more substantially improve their reading skills. **Another remaining issue is whether the observed positive effects at the end of trainings would lead to long-term progress in reading after the end of any special remediation.** Last, we should expect each of the RapDys© and MAEVA© training programs to more efficiently improve reading performance if proposed to subgroups of children with dyslexia with specific phonological or VA span deficits, respectively.

## Ethics Statement

This study was carried out in accordance with the Declaration of Helsinki. Written informed consent was obtained from the legal tutor of all participants. The protocol was approved by the Ethic Committee of the University Grenoble-Alpes (CERNI).

## Author Contributions

RZ designed the intervention study, participated to the collection and analysis of the data as part of her Ph.D., and to the writing of the manuscript. GC, SV, and WS participated to design of the study, to data analyses, and to the writing of the manuscript. M-AN-M participated to design of the study and to the collection of the data.

## Conflict of Interest Statement

GC and WS declare a conflict of interest as they might benefit from the diffusion of the RapDys© method by SATT-INNOV IDF (ERGANEO). The remaining authors declare that the research was conducted in the absence of any commercial or financial relationships that could be construed as a potential conflict of interest.

## Footnotes

1 We were not able to assess the effects of these two training programs in different subgroups of children with either a unique categorical perception deficit or a unique VA span deficit because of the small number of participants with a selective deficit. Around 30% of individuals with developmental dyslexia were reported to have a selective deficit which would concern around 15 of our participants, who should be further divided in two matched groups.

2 VOT is the time interval between the release from stop closure and the onset of laryngeal pulsing (Lisker and Abramson, 1964). VOT is a major acoustic cue for the perception of the voicing feature that separates the /b d g/ from the /ptk/ stop consonants (in English: Lisker et al., 1977; in French: Serniclaes, 1987). Voiced stops display a negative VOT (laryngeal pulsing starts before closure release), voiceless stops display a positive VOT (laryngeal pulsing starts after closure release).

## Supplementary Material

TABLE S1 Numerical simulations showing that orthogonal contrasts are not correlated.
